# Crystal structure of bis­(acetonyltri­phenyl­phospho­nium) tetra­chlorido­cobaltate(II)

**DOI:** 10.1107/S2056989015019180

**Published:** 2015-11-04

**Authors:** Mouhamadou Birame Diop, Libasse Diop, Allen G. Oliver

**Affiliations:** a Laboratoire de Chimie Minérale et Analytique, Département de Chimie, Faculté des Sciences et Techniques, Université Cheikh Anta Diop, Dakar, Senegal; b Department of Chemistry and Biochemistry, University of Notre Dame, 246 Nieuwland Science Hall, Notre Dame, IN 46557-5670, USA

**Keywords:** crystal structure, tetra­chlorido­cobaltate dianion, acetonyltri­phenyl­phospho­nium cation, alkyl­tri­phenyl­phospho­nium

## Abstract

The complex title salt, (C_21_H_20_OP)_2_[CoCl_4_], is the reaction product of CoCl_2_ with acetonyltri­phenyl­phospho­nium chloride in aceto­nitrile. In the anion, the Co^II^ atom exhibits a typical tetra­hedral environment, with Co—Cl distances ranging from 2.2721 (6) to 2.2901 (6) Å, and with Cl—Co—Cl angles ranging from 106.12 (2) to 112.24 (2)°. The two phospho­nium cations likewise show the expected tetra­hedral configuration, with P—C distances ranging from 1.785 (2) to 1.8059 (18) Å and C—P—C angles ranging from 106.98 (8) to 112.85 (15)°. The mol­ecules inter­act in the lattice mainly through Coulombic and van der Waals forces because there is no particular polarity to the charges carried by the cations or anion. In the crystal, the cations and anions are arranged in sheets parallel to (001).

## Related literature   

Cobalt(II) and cobalt(III) compounds can show a variety of extended structural arrangements and are used as metal catalysts (Adams *et al.*, 2008 ▸; Boudraa *et al.*, 2015 ▸; Bronova *et al.*, 2013 ▸; Dhieb *et al.*, 2014 ▸; Lassahn *et al.*, 2003 ▸; Luo *et al.*, 2013 ▸; Merola *et al.*, 2013 ▸). Alkyl­tri­phenyl­phospho­nium cations have been employed as stabilizing cations for a variety of different anions, such as nitrate, tetra­phenyl­borate and bromide (Diop *et al.*, 2013 ▸; Evans, 2010 ▸; Kavitha *et al.*, 2012 ▸). For other structures containing the tetra­hedral [CoCl_4_]^2−^ anion, see: Diop *et al.* (2015 ▸); Gueddar *et al.* (2013 ▸).
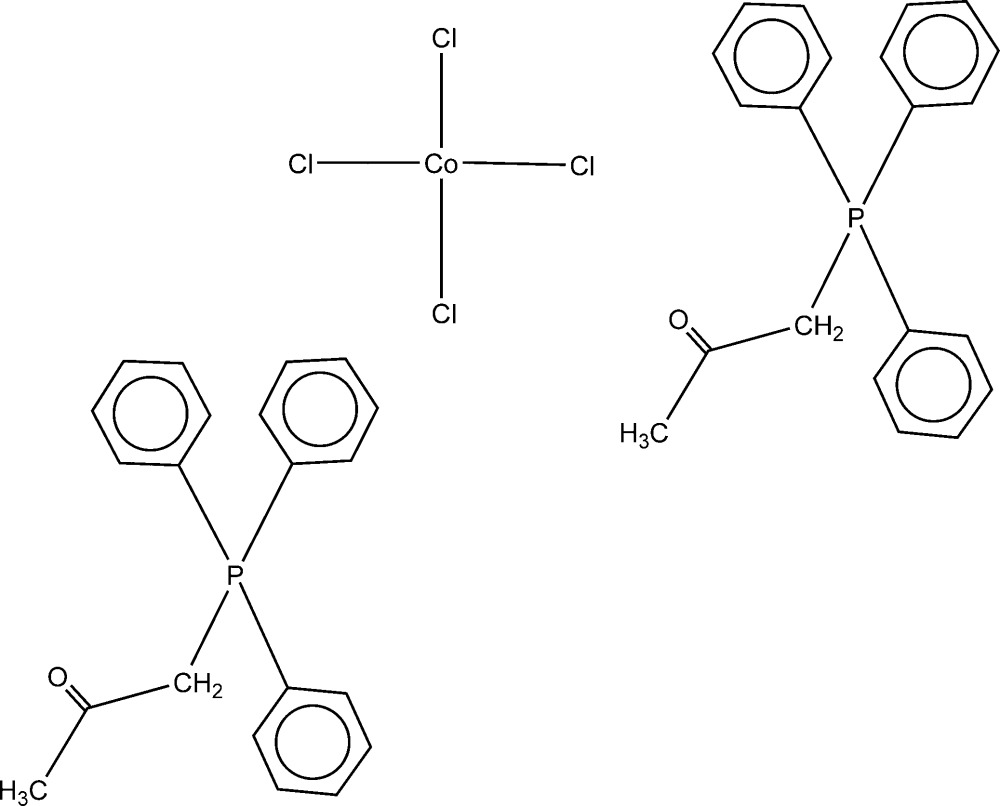


## Experimental   

### Crystal data  

(C_21_H_20_OP)_2_[CoCl_4_]
*M* *_r_* = 839.41
Orthorhombic, 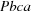

*a* = 18.758 (3) Å
*b* = 15.769 (2) Å
*c* = 27.157 (4) Å
*V* = 8033 (2) Å^3^
*Z* = 8
Mo *K*α radiation
μ = 0.81 mm^−1^
*T* = 120 K
0.24 × 0.16 × 0.10 mm

### Data collection  

Bruker Kappa X8-APEXII diffractometer
Absorption correction: numerical (*SADABS*; Krause *et al.*, 2015 ▸) *T*_min_ = 0.813, *T*_max_ = 0.920
148135 measured reflections
10073 independent reflections
8207 reflections with *I* > 2σ(*I*)
*R*_int_ = 0.061

### Refinement  

*R*[*F* ^2^ > 2σ(*F* ^2^)] = 0.038
*wR*(*F* ^2^) = 0.097
*S* = 1.06
10073 reflections
462 parameters
H-atom parameters constrained
Δρ_max_ = 0.76 e Å^−3^
Δρ_min_ = −0.25 e Å^−3^

### 

Data collection: *APEX2* (Bruker, 2014 ▸); cell refinement: *SAINT* (Bruker, 2014 ▸); data reduction: *SAINT*; program(s) used to solve structure: *SHELXT* (Sheldrick, 2015*a* ▸); program(s) used to refine structure: *SHELXL2014* (Sheldrick, 2015*b* ▸); molecular graphics: *XP* in *SHELXTL* (Sheldrick, 2008 ▸) and *Mercury* (Macrae *et al.*, 2006 ▸); software used to prepare material for publication: *CIFTAB* (Sheldrick, 2008 ▸) and *publCIF* (Westrip, 2010 ▸).

## Supplementary Material

Crystal structure: contains datablock(s) I. DOI: 10.1107/S2056989015019180/wm5224sup1.cif

Structure factors: contains datablock(s) I. DOI: 10.1107/S2056989015019180/wm5224Isup2.hkl

**Fig. 1.**
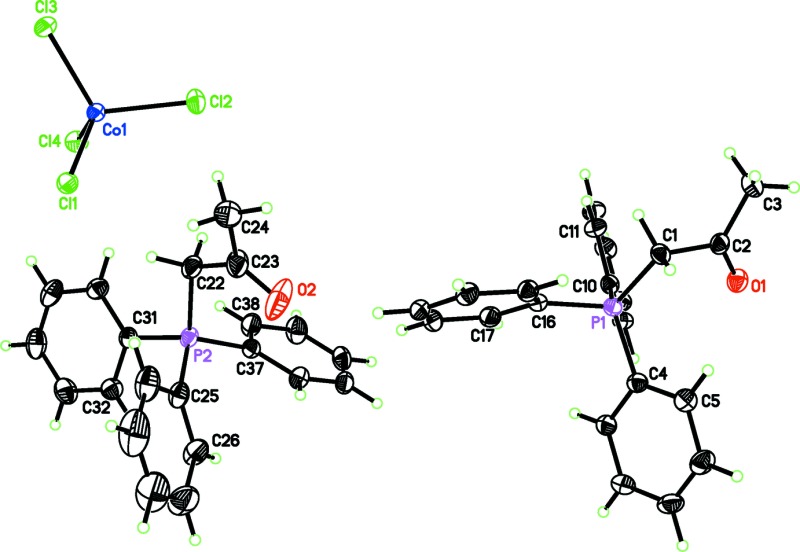
The molecular components of the title compound. Displacement ellipsoids are drawn at the 50% probability level.

**Fig. 2.**
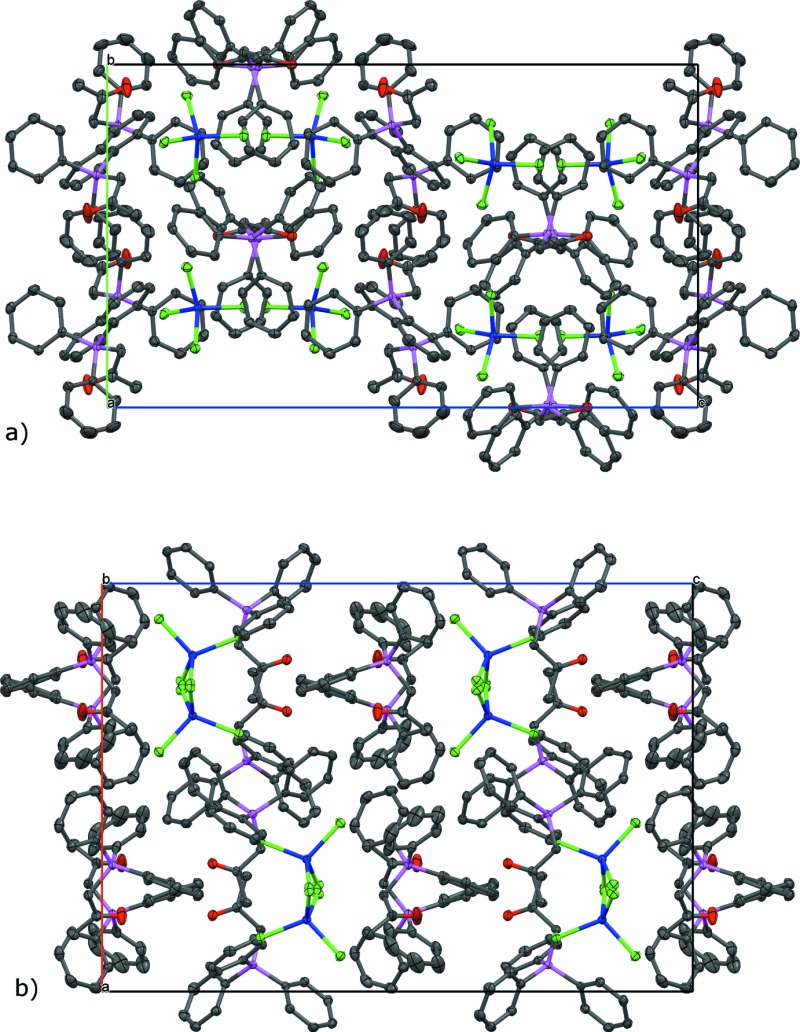
Packing views of the title compound a) along [100] and b) along [010]. Hydrogen atoms were omitted for clarity. Displacement ellipsoids are drawn at the 50% probability level.

. DOI: 10.1107/S2056989015019180/wm5224fig1.tif
The mol­ecular components of the title compound. Displacement ellipsoids are drawn at the 50% probability level.

a b . DOI: 10.1107/S2056989015019180/wm5224fig2.tif
Packing views of the title compound *a*) along [100] and *b*) along [010]. Hydrogen atoms were omitted for clarity. Displacement ellipsoids are drawn at the 50% probability level.

CCDC reference: 1430699

Additional supporting information:  crystallographic information; 3D view; checkCIF report

## supplementary crystallographic information

### S1. Synthesis and crystallization

All chemicals were purchased from Aldrich-Germany and were used as received. Acetonyl tri­phenyl­phospho­nium chloride was mixed in aceto­nitrile with CoCl_2_·6H_2_O in a 2:1 ratio. Blue crystals suitable for a single-crystal X-ray diffraction study were obtained after slow solvent evaporation at room temperature.

### S2. Refinement

Hydrogen atoms were included in idealized positions with *U*_iso_(H) = 1.2*U*_eq_(C_aromatic/methyl­ene_) or 1.5*U*_eq_(C_methyl_). C—H distances were set to 0.95 (aromatic), 0.98 (methyl) and 0.99 Å (methyl­ene).

### Crystal data

**Table:**

(C_21_H_20_OP)_2_[CoCl_4_]	*D*_x_ = 1.388 Mg m^−^^3^
*M**_r_* = 839.41	Mo *K*α radiation, λ = 0.71073 Å
Orthorhombic, *P**b**c**a*	Cell parameters from 9962 reflections
*a* = 18.758 (3) Å	θ = 2.3–28.3°
*b* = 15.769 (2) Å	µ = 0.81 mm^−^^1^
*c* = 27.157 (4) Å	*T* = 120 K
*V* = 8033 (2) Å^3^	Tablet, blue
*Z* = 8	0.24 × 0.16 × 0.10 mm
*F*(000) = 3464	

### Data collection

**Table:**

Bruker Kappa X8-APEXII diffractometer	10073 independent reflections
Radiation source: fine-focus sealed tube	8207 reflections with *I* > 2σ(*I*)
Graphite monochromator	*R*_int_ = 0.061
Detector resolution: 8.33 pixels mm^-1^	θ_max_ = 28.4°, θ_min_ = 1.5°
combination of ω and φ–scans	*h* = −25→25
Absorption correction: numerical (*SADABS*; Krause *et al.*, 2015)	*k* = −21→21
*T*_min_ = 0.813, *T*_max_ = 0.920	*l* = −36→36
148135 measured reflections	

### Refinement

**Table:**

Refinement on *F*^2^	Primary atom site location: real-space vector search
Least-squares matrix: full	Secondary atom site location: difference Fourier map
*R*[*F*^2^ > 2σ(*F*^2^)] = 0.038	Hydrogen site location: inferred from neighbouring sites
*wR*(*F*^2^) = 0.097	H-atom parameters constrained
*S* = 1.06	*w* = 1/[σ^2^(*F*_o_^2^) + (0.047*P*)^2^ + 5.1834*P*] where *P* = (*F*_o_^2^ + 2*F*_c_^2^)/3
10073 reflections	(Δ/σ)_max_ = 0.002
462 parameters	Δρ_max_ = 0.76 e Å^−^^3^
0 restraints	Δρ_min_ = −0.25 e Å^−^^3^

### Special details

**Table:**

Geometry. All e.s.d.'s (except the e.s.d. in the dihedral angle between two l.s. planes) are estimated using the full covariance matrix. The cell e.s.d.'s are taken into account individually in the estimation of e.s.d.'s in distances, angles and torsion angles; correlations between e.s.d.'s in cell parameters are only used when they are defined by crystal symmetry. An approximate (isotropic) treatment of cell e.s.d.'s is used for estimating e.s.d.'s involving l.s. planes.

### Fractional atomic coordinates and isotropic or equivalent isotropic displacement parameters (Å^2^)

**Table:**

	*x*	*y*	*z*	*U*_iso_*/*U*_eq_	
Co1	0.67585 (2)	0.78995 (2)	0.34640 (2)	0.01815 (7)	
Cl1	0.74555 (3)	0.67130 (3)	0.35096 (2)	0.02314 (10)	
Cl2	0.58678 (3)	0.77319 (3)	0.40325 (2)	0.02611 (11)	
Cl3	0.63163 (3)	0.79320 (3)	0.26854 (2)	0.02741 (11)	
Cl4	0.73968 (3)	0.90911 (3)	0.36500 (2)	0.02644 (11)	
P1	0.43467 (2)	0.50603 (3)	0.74508 (2)	0.01730 (10)	
O1	0.31362 (7)	0.50028 (9)	0.81285 (5)	0.0253 (3)	
C1	0.34791 (9)	0.46217 (12)	0.73157 (7)	0.0205 (4)	
H1A	0.3308	0.4851	0.6998	0.025*	
H1B	0.3520	0.3998	0.7282	0.025*	
C2	0.29400 (10)	0.48277 (11)	0.77155 (7)	0.0205 (4)	
C3	0.21765 (10)	0.48012 (13)	0.75622 (8)	0.0268 (4)	
H3A	0.2090	0.4289	0.7367	0.040*	
H3B	0.2066	0.5304	0.7364	0.040*	
H3C	0.1872	0.4794	0.7855	0.040*	
C4	0.48127 (10)	0.44742 (12)	0.79153 (7)	0.0201 (4)	
C5	0.45435 (11)	0.37160 (12)	0.80994 (7)	0.0243 (4)	
H5	0.4095	0.3509	0.7990	0.029*	
C6	0.49371 (12)	0.32628 (13)	0.84449 (8)	0.0293 (4)	
H6	0.4757	0.2743	0.8571	0.035*	
C7	0.55904 (12)	0.35646 (13)	0.86060 (8)	0.0295 (4)	
H7	0.5853	0.3254	0.8845	0.035*	
C8	0.58631 (11)	0.43149 (13)	0.84208 (7)	0.0261 (4)	
H8	0.6311	0.4519	0.8533	0.031*	
C9	0.54805 (10)	0.47700 (12)	0.80697 (7)	0.0228 (4)	
H9	0.5671	0.5278	0.7936	0.027*	
C10	0.42343 (9)	0.61523 (11)	0.76149 (7)	0.0189 (3)	
C11	0.38399 (11)	0.66634 (13)	0.72936 (7)	0.0244 (4)	
H11	0.3648	0.6432	0.6999	0.029*	
C12	0.37310 (11)	0.75082 (13)	0.74080 (8)	0.0281 (4)	
H12	0.3460	0.7857	0.7193	0.034*	
C13	0.40164 (11)	0.78474 (13)	0.78367 (8)	0.0269 (4)	
H13	0.3949	0.8431	0.7911	0.032*	
C14	0.43982 (11)	0.73370 (13)	0.81562 (7)	0.0256 (4)	
H14	0.4588	0.7571	0.8451	0.031*	
C15	0.45054 (10)	0.64892 (12)	0.80503 (7)	0.0217 (4)	
H15	0.4762	0.6139	0.8273	0.026*	
C16	0.48683 (9)	0.49899 (12)	0.68965 (6)	0.0187 (3)	
C17	0.54246 (10)	0.55695 (13)	0.68207 (7)	0.0233 (4)	
H17	0.5488	0.6035	0.7039	0.028*	
C18	0.58806 (10)	0.54562 (14)	0.64240 (7)	0.0264 (4)	
H18	0.6261	0.5844	0.6371	0.032*	
C19	0.57847 (11)	0.47806 (13)	0.61039 (7)	0.0256 (4)	
H19	0.6102	0.4704	0.5835	0.031*	
C20	0.52278 (11)	0.42182 (12)	0.61749 (7)	0.0235 (4)	
H20	0.5158	0.3764	0.5950	0.028*	
C21	0.47709 (10)	0.43131 (12)	0.65729 (7)	0.0215 (4)	
H21	0.4394	0.3920	0.6625	0.026*	
P2	0.81009 (3)	0.67681 (3)	0.51792 (2)	0.02010 (10)	
O2	0.68507 (12)	0.56656 (15)	0.53292 (6)	0.0650 (7)	
C22	0.73531 (11)	0.66612 (14)	0.47682 (7)	0.0291 (4)	
H22A	0.7082	0.7199	0.4765	0.035*	
H22B	0.7532	0.6559	0.4430	0.035*	
C23	0.68560 (12)	0.59424 (16)	0.49123 (8)	0.0339 (5)	
C24	0.64020 (12)	0.55970 (15)	0.45136 (8)	0.0338 (5)	
H24A	0.6195	0.6066	0.4325	0.051*	
H24B	0.6019	0.5254	0.4657	0.051*	
H24C	0.6692	0.5242	0.4295	0.051*	
C25	0.85355 (11)	0.57674 (12)	0.52453 (7)	0.0257 (4)	
C26	0.89525 (13)	0.56012 (14)	0.56578 (8)	0.0346 (5)	
H26	0.8976	0.6002	0.5918	0.041*	
C27	0.93322 (17)	0.48528 (18)	0.56879 (10)	0.0547 (8)	
H27	0.9618	0.4738	0.5969	0.066*	
C28	0.9295 (2)	0.42738 (18)	0.53091 (12)	0.0666 (10)	
H28	0.9554	0.3758	0.5332	0.080*	
C29	0.8887 (2)	0.44340 (17)	0.48981 (11)	0.0613 (9)	
H29	0.8866	0.4029	0.4640	0.074*	
C30	0.85083 (15)	0.51835 (15)	0.48610 (9)	0.0396 (6)	
H30	0.8232	0.5299	0.4576	0.048*	
C31	0.87237 (10)	0.74834 (12)	0.49010 (7)	0.0207 (4)	
C32	0.94058 (11)	0.75279 (13)	0.51041 (7)	0.0262 (4)	
H32	0.9520	0.7204	0.5388	0.031*	
C33	0.99159 (12)	0.80463 (15)	0.48902 (9)	0.0341 (5)	
H33	1.0381	0.8078	0.5027	0.041*	
C34	0.97468 (13)	0.85188 (14)	0.44761 (8)	0.0361 (5)	
H34	1.0099	0.8869	0.4328	0.043*	
C35	0.90704 (13)	0.84835 (13)	0.42767 (8)	0.0329 (5)	
H35	0.8959	0.8815	0.3995	0.039*	
C36	0.85523 (11)	0.79668 (12)	0.44854 (7)	0.0251 (4)	
H36	0.8087	0.7942	0.4348	0.030*	
C37	0.78322 (10)	0.71941 (12)	0.57609 (7)	0.0208 (4)	
C38	0.77556 (12)	0.80730 (13)	0.58000 (8)	0.0294 (4)	
H38	0.7870	0.8429	0.5529	0.035*	
C39	0.75113 (13)	0.84223 (14)	0.62383 (8)	0.0348 (5)	
H39	0.7453	0.9019	0.6267	0.042*	
C40	0.73537 (11)	0.79010 (14)	0.66319 (8)	0.0290 (4)	
H40	0.7190	0.8143	0.6932	0.035*	
C41	0.74312 (11)	0.70303 (13)	0.65961 (7)	0.0257 (4)	
H41	0.7325	0.6679	0.6871	0.031*	
C42	0.76634 (10)	0.66707 (12)	0.61586 (7)	0.0237 (4)	
H42	0.7707	0.6073	0.6130	0.028*	

### Atomic displacement parameters (Å^2^)

**Table:**

	*U*^11^	*U*^22^	*U*^33^	*U*^12^	*U*^13^	*U*^23^
Co1	0.01844 (13)	0.01928 (13)	0.01673 (12)	0.00064 (9)	−0.00024 (9)	−0.00037 (9)
Cl1	0.0255 (2)	0.0228 (2)	0.0212 (2)	0.00573 (18)	0.00104 (17)	−0.00089 (16)
Cl2	0.0239 (2)	0.0303 (2)	0.0241 (2)	−0.00433 (19)	0.00580 (18)	−0.00585 (18)
Cl3	0.0284 (2)	0.0360 (3)	0.0178 (2)	0.0041 (2)	−0.00300 (17)	0.00000 (18)
Cl4	0.0277 (2)	0.0213 (2)	0.0303 (2)	−0.00336 (18)	−0.00315 (19)	0.00061 (18)
P1	0.0163 (2)	0.0186 (2)	0.0170 (2)	−0.00126 (17)	−0.00101 (17)	−0.00021 (17)
O1	0.0276 (7)	0.0272 (7)	0.0210 (7)	−0.0022 (6)	0.0019 (5)	−0.0008 (5)
C1	0.0171 (8)	0.0241 (9)	0.0203 (9)	−0.0038 (7)	−0.0007 (7)	−0.0018 (7)
C2	0.0221 (9)	0.0166 (8)	0.0229 (9)	−0.0008 (7)	0.0018 (7)	0.0037 (7)
C3	0.0198 (9)	0.0299 (10)	0.0308 (10)	−0.0007 (8)	0.0028 (8)	−0.0013 (8)
C4	0.0207 (9)	0.0203 (9)	0.0192 (8)	0.0020 (7)	−0.0008 (7)	−0.0005 (7)
C5	0.0253 (9)	0.0210 (9)	0.0266 (10)	−0.0001 (8)	0.0000 (8)	−0.0007 (8)
C6	0.0364 (12)	0.0196 (9)	0.0320 (11)	0.0028 (8)	0.0007 (9)	0.0058 (8)
C7	0.0320 (11)	0.0278 (10)	0.0288 (10)	0.0105 (9)	−0.0038 (8)	0.0041 (8)
C8	0.0216 (9)	0.0284 (10)	0.0285 (10)	0.0027 (8)	−0.0041 (8)	−0.0011 (8)
C9	0.0208 (9)	0.0226 (9)	0.0250 (9)	0.0009 (7)	0.0005 (7)	0.0023 (7)
C10	0.0182 (8)	0.0172 (8)	0.0213 (9)	−0.0006 (7)	0.0013 (7)	0.0001 (7)
C11	0.0259 (10)	0.0271 (10)	0.0201 (9)	0.0012 (8)	−0.0043 (7)	0.0013 (7)
C12	0.0296 (11)	0.0251 (10)	0.0295 (10)	0.0054 (8)	−0.0028 (8)	0.0060 (8)
C13	0.0251 (10)	0.0197 (9)	0.0358 (11)	0.0014 (8)	0.0010 (8)	0.0003 (8)
C14	0.0248 (10)	0.0247 (10)	0.0272 (10)	0.0001 (8)	−0.0046 (8)	−0.0053 (8)
C15	0.0200 (9)	0.0222 (9)	0.0229 (9)	0.0015 (7)	−0.0037 (7)	0.0014 (7)
C16	0.0173 (8)	0.0224 (9)	0.0165 (8)	0.0017 (7)	−0.0009 (7)	0.0021 (7)
C17	0.0221 (9)	0.0262 (10)	0.0215 (9)	−0.0040 (8)	−0.0033 (7)	0.0010 (7)
C18	0.0190 (9)	0.0330 (11)	0.0273 (10)	−0.0055 (8)	0.0002 (7)	0.0059 (8)
C19	0.0231 (10)	0.0330 (11)	0.0209 (9)	0.0066 (8)	0.0032 (7)	0.0075 (8)
C20	0.0287 (10)	0.0227 (9)	0.0192 (9)	0.0044 (8)	0.0010 (7)	−0.0012 (7)
C21	0.0226 (9)	0.0206 (9)	0.0214 (9)	−0.0014 (7)	−0.0001 (7)	0.0016 (7)
P2	0.0219 (2)	0.0232 (2)	0.0152 (2)	−0.00295 (19)	−0.00015 (17)	−0.00036 (18)
O2	0.0754 (14)	0.0948 (16)	0.0248 (9)	−0.0567 (13)	−0.0012 (9)	0.0086 (9)
C22	0.0260 (10)	0.0428 (12)	0.0185 (9)	−0.0090 (9)	−0.0017 (8)	0.0017 (8)
C23	0.0287 (11)	0.0486 (13)	0.0244 (10)	−0.0140 (10)	0.0014 (8)	−0.0022 (9)
C24	0.0311 (11)	0.0371 (12)	0.0333 (11)	−0.0074 (9)	−0.0039 (9)	−0.0041 (9)
C25	0.0348 (11)	0.0201 (9)	0.0222 (9)	−0.0018 (8)	0.0061 (8)	−0.0016 (7)
C26	0.0506 (14)	0.0274 (11)	0.0257 (10)	0.0107 (10)	0.0044 (10)	0.0005 (8)
C27	0.076 (2)	0.0447 (15)	0.0433 (15)	0.0297 (14)	0.0098 (14)	0.0113 (12)
C28	0.098 (3)	0.0351 (14)	0.067 (2)	0.0279 (16)	0.0277 (19)	0.0062 (14)
C29	0.097 (2)	0.0298 (13)	0.0571 (18)	−0.0002 (15)	0.0307 (17)	−0.0183 (12)
C30	0.0544 (15)	0.0327 (12)	0.0318 (12)	−0.0071 (11)	0.0118 (11)	−0.0096 (9)
C31	0.0231 (9)	0.0201 (9)	0.0187 (8)	−0.0006 (7)	0.0024 (7)	−0.0022 (7)
C32	0.0251 (10)	0.0263 (10)	0.0272 (10)	−0.0025 (8)	−0.0019 (8)	0.0007 (8)
C33	0.0257 (11)	0.0365 (12)	0.0400 (12)	−0.0076 (9)	0.0024 (9)	−0.0040 (10)
C34	0.0423 (13)	0.0297 (11)	0.0364 (12)	−0.0119 (10)	0.0138 (10)	−0.0011 (9)
C35	0.0504 (14)	0.0245 (10)	0.0237 (10)	−0.0044 (9)	0.0043 (9)	0.0030 (8)
C36	0.0304 (10)	0.0225 (9)	0.0223 (9)	0.0012 (8)	−0.0015 (8)	−0.0005 (7)
C37	0.0198 (9)	0.0249 (9)	0.0176 (8)	0.0003 (7)	0.0003 (7)	−0.0010 (7)
C38	0.0345 (11)	0.0275 (10)	0.0263 (10)	0.0039 (9)	0.0065 (8)	0.0033 (8)
C39	0.0421 (13)	0.0268 (11)	0.0355 (12)	0.0041 (9)	0.0116 (10)	−0.0046 (9)
C40	0.0275 (11)	0.0341 (11)	0.0255 (10)	0.0012 (9)	0.0076 (8)	−0.0063 (8)
C41	0.0250 (10)	0.0320 (11)	0.0202 (9)	−0.0034 (8)	0.0031 (8)	0.0001 (8)
C42	0.0274 (10)	0.0234 (9)	0.0202 (9)	−0.0022 (8)	0.0011 (7)	−0.0004 (7)

### Geometric parameters (Å, º)

**Table:**

Co1—Cl3	2.2721 (6)	C20—H20	0.9500
Co1—Cl4	2.2845 (6)	C21—H21	0.9500
Co1—Cl1	2.2859 (6)	P2—C25	1.785 (2)
Co1—Cl2	2.2901 (6)	P2—C37	1.7892 (19)
P1—C10	1.7912 (19)	P2—C31	1.7912 (19)
P1—C4	1.7914 (19)	P2—C22	1.801 (2)
P1—C16	1.7988 (18)	O2—C23	1.213 (3)
P1—C1	1.8059 (18)	C22—C23	1.519 (3)
O1—C2	1.212 (2)	C22—H22A	0.9900
C1—C2	1.519 (3)	C22—H22B	0.9900
C1—H1A	0.9900	C23—C24	1.481 (3)
C1—H1B	0.9900	C24—H24A	0.9800
C2—C3	1.492 (3)	C24—H24B	0.9800
C3—H3A	0.9800	C24—H24C	0.9800
C3—H3B	0.9800	C25—C26	1.391 (3)
C3—H3C	0.9800	C25—C30	1.393 (3)
C4—C5	1.391 (3)	C26—C27	1.381 (3)
C4—C9	1.401 (3)	C26—H26	0.9500
C5—C6	1.391 (3)	C27—C28	1.377 (4)
C5—H5	0.9500	C27—H27	0.9500
C6—C7	1.386 (3)	C28—C29	1.377 (5)
C6—H6	0.9500	C28—H28	0.9500
C7—C8	1.384 (3)	C29—C30	1.382 (4)
C7—H7	0.9500	C29—H29	0.9500
C8—C9	1.392 (3)	C30—H30	0.9500
C8—H8	0.9500	C31—C32	1.395 (3)
C9—H9	0.9500	C31—C36	1.399 (3)
C10—C15	1.392 (3)	C32—C33	1.386 (3)
C10—C11	1.399 (3)	C32—H32	0.9500
C11—C12	1.383 (3)	C33—C34	1.386 (3)
C11—H11	0.9500	C33—H33	0.9500
C12—C13	1.389 (3)	C34—C35	1.381 (3)
C12—H12	0.9500	C34—H34	0.9500
C13—C14	1.383 (3)	C35—C36	1.389 (3)
C13—H13	0.9500	C35—H35	0.9500
C14—C15	1.382 (3)	C36—H36	0.9500
C14—H14	0.9500	C37—C42	1.396 (3)
C15—H15	0.9500	C37—C38	1.397 (3)
C16—C21	1.394 (3)	C38—C39	1.389 (3)
C16—C17	1.402 (3)	C38—H38	0.9500
C17—C18	1.387 (3)	C39—C40	1.380 (3)
C17—H17	0.9500	C39—H39	0.9500
C18—C19	1.387 (3)	C40—C41	1.384 (3)
C18—H18	0.9500	C40—H40	0.9500
C19—C20	1.384 (3)	C41—C42	1.387 (3)
C19—H19	0.9500	C41—H41	0.9500
C20—C21	1.387 (3)	C42—H42	0.9500
			
Cl3—Co1—Cl4	112.24 (2)	C20—C21—C16	119.51 (18)
Cl3—Co1—Cl1	106.12 (2)	C20—C21—H21	120.2
Cl4—Co1—Cl1	111.19 (2)	C16—C21—H21	120.2
Cl3—Co1—Cl2	111.32 (2)	C25—P2—C37	111.83 (9)
Cl4—Co1—Cl2	109.17 (2)	C25—P2—C31	107.53 (9)
Cl1—Co1—Cl2	106.64 (2)	C37—P2—C31	108.65 (9)
C10—P1—C4	112.22 (8)	C25—P2—C22	109.59 (10)
C10—P1—C16	109.37 (9)	C37—P2—C22	111.29 (10)
C4—P1—C16	106.98 (8)	C31—P2—C22	107.79 (9)
C10—P1—C1	108.21 (9)	C23—C22—P2	112.85 (15)
C4—P1—C1	112.66 (9)	C23—C22—H22A	109.0
C16—P1—C1	107.25 (8)	P2—C22—H22A	109.0
C2—C1—P1	111.90 (13)	C23—C22—H22B	109.0
C2—C1—H1A	109.2	P2—C22—H22B	109.0
P1—C1—H1A	109.2	H22A—C22—H22B	107.8
C2—C1—H1B	109.2	O2—C23—C24	123.0 (2)
P1—C1—H1B	109.2	O2—C23—C22	120.91 (19)
H1A—C1—H1B	107.9	C24—C23—C22	116.03 (18)
O1—C2—C3	123.76 (18)	C23—C24—H24A	109.5
O1—C2—C1	120.53 (17)	C23—C24—H24B	109.5
C3—C2—C1	115.71 (16)	H24A—C24—H24B	109.5
C2—C3—H3A	109.5	C23—C24—H24C	109.5
C2—C3—H3B	109.5	H24A—C24—H24C	109.5
H3A—C3—H3B	109.5	H24B—C24—H24C	109.5
C2—C3—H3C	109.5	C26—C25—C30	120.0 (2)
H3A—C3—H3C	109.5	C26—C25—P2	120.28 (15)
H3B—C3—H3C	109.5	C30—C25—P2	119.50 (18)
C5—C4—C9	120.21 (17)	C27—C26—C25	119.9 (2)
C5—C4—P1	121.30 (14)	C27—C26—H26	120.0
C9—C4—P1	118.38 (14)	C25—C26—H26	120.0
C4—C5—C6	119.42 (19)	C28—C27—C26	119.7 (3)
C4—C5—H5	120.3	C28—C27—H27	120.1
C6—C5—H5	120.3	C26—C27—H27	120.1
C7—C6—C5	120.39 (19)	C29—C28—C27	120.8 (3)
C7—C6—H6	119.8	C29—C28—H28	119.6
C5—C6—H6	119.8	C27—C28—H28	119.6
C8—C7—C6	120.40 (19)	C28—C29—C30	120.1 (2)
C8—C7—H7	119.8	C28—C29—H29	120.0
C6—C7—H7	119.8	C30—C29—H29	120.0
C7—C8—C9	119.93 (19)	C29—C30—C25	119.5 (3)
C7—C8—H8	120.0	C29—C30—H30	120.3
C9—C8—H8	120.0	C25—C30—H30	120.3
C8—C9—C4	119.62 (18)	C32—C31—C36	120.16 (18)
C8—C9—H9	120.2	C32—C31—P2	117.58 (14)
C4—C9—H9	120.2	C36—C31—P2	122.23 (15)
C15—C10—C11	120.19 (17)	C33—C32—C31	119.79 (19)
C15—C10—P1	122.35 (14)	C33—C32—H32	120.1
C11—C10—P1	117.43 (14)	C31—C32—H32	120.1
C12—C11—C10	119.52 (18)	C34—C33—C32	120.0 (2)
C12—C11—H11	120.2	C34—C33—H33	120.0
C10—C11—H11	120.2	C32—C33—H33	120.0
C11—C12—C13	120.16 (18)	C35—C34—C33	120.5 (2)
C11—C12—H12	119.9	C35—C34—H34	119.8
C13—C12—H12	119.9	C33—C34—H34	119.8
C14—C13—C12	120.10 (19)	C34—C35—C36	120.4 (2)
C14—C13—H13	120.0	C34—C35—H35	119.8
C12—C13—H13	120.0	C36—C35—H35	119.8
C15—C14—C13	120.50 (18)	C35—C36—C31	119.20 (19)
C15—C14—H14	119.8	C35—C36—H36	120.4
C13—C14—H14	119.8	C31—C36—H36	120.4
C14—C15—C10	119.51 (17)	C42—C37—C38	120.29 (18)
C14—C15—H15	120.2	C42—C37—P2	121.68 (15)
C10—C15—H15	120.2	C38—C37—P2	117.94 (14)
C21—C16—C17	120.25 (17)	C39—C38—C37	119.49 (19)
C21—C16—P1	120.22 (14)	C39—C38—H38	120.3
C17—C16—P1	119.17 (14)	C37—C38—H38	120.3
C18—C17—C16	119.27 (18)	C40—C39—C38	119.9 (2)
C18—C17—H17	120.4	C40—C39—H39	120.1
C16—C17—H17	120.4	C38—C39—H39	120.1
C19—C18—C17	120.39 (18)	C39—C40—C41	120.92 (19)
C19—C18—H18	119.8	C39—C40—H40	119.5
C17—C18—H18	119.8	C41—C40—H40	119.5
C20—C19—C18	120.18 (18)	C40—C41—C42	119.92 (19)
C20—C19—H19	119.9	C40—C41—H41	120.0
C18—C19—H19	119.9	C42—C41—H41	120.0
C19—C20—C21	120.39 (18)	C41—C42—C37	119.50 (18)
C19—C20—H20	119.8	C41—C42—H42	120.2
C21—C20—H20	119.8	C37—C42—H42	120.2
			
C10—P1—C1—C2	−48.98 (15)	C25—P2—C22—C23	51.93 (18)
C4—P1—C1—C2	75.68 (15)	C37—P2—C22—C23	−72.28 (18)
C16—P1—C1—C2	−166.87 (13)	C31—P2—C22—C23	168.68 (15)
P1—C1—C2—O1	−22.6 (2)	P2—C22—C23—O2	21.0 (3)
P1—C1—C2—C3	157.19 (14)	P2—C22—C23—C24	−157.50 (18)
C10—P1—C4—C5	129.00 (16)	C37—P2—C25—C26	−33.4 (2)
C16—P1—C4—C5	−111.05 (16)	C31—P2—C25—C26	85.80 (19)
C1—P1—C4—C5	6.56 (19)	C22—P2—C25—C26	−157.29 (18)
C10—P1—C4—C9	−54.84 (17)	C37—P2—C25—C30	152.48 (17)
C16—P1—C4—C9	65.11 (16)	C31—P2—C25—C30	−88.33 (19)
C1—P1—C4—C9	−177.28 (14)	C22—P2—C25—C30	28.6 (2)
C9—C4—C5—C6	1.2 (3)	C30—C25—C26—C27	−0.8 (4)
P1—C4—C5—C6	177.30 (15)	P2—C25—C26—C27	−174.9 (2)
C4—C5—C6—C7	0.2 (3)	C25—C26—C27—C28	−0.1 (4)
C5—C6—C7—C8	−0.8 (3)	C26—C27—C28—C29	0.5 (5)
C6—C7—C8—C9	−0.1 (3)	C27—C28—C29—C30	0.0 (5)
C7—C8—C9—C4	1.6 (3)	C28—C29—C30—C25	−0.9 (4)
C5—C4—C9—C8	−2.1 (3)	C26—C25—C30—C29	1.2 (4)
P1—C4—C9—C8	−178.31 (15)	P2—C25—C30—C29	175.4 (2)
C4—P1—C10—C15	1.85 (18)	C25—P2—C31—C32	−49.08 (17)
C16—P1—C10—C15	−116.70 (16)	C37—P2—C31—C32	72.13 (17)
C1—P1—C10—C15	126.77 (16)	C22—P2—C31—C32	−167.16 (15)
C4—P1—C10—C11	−176.27 (14)	C25—P2—C31—C36	128.93 (16)
C16—P1—C10—C11	65.18 (16)	C37—P2—C31—C36	−109.86 (16)
C1—P1—C10—C11	−51.35 (17)	C22—P2—C31—C36	10.86 (19)
C15—C10—C11—C12	1.0 (3)	C36—C31—C32—C33	−0.6 (3)
P1—C10—C11—C12	179.16 (16)	P2—C31—C32—C33	177.42 (16)
C10—C11—C12—C13	0.5 (3)	C31—C32—C33—C34	0.1 (3)
C11—C12—C13—C14	−1.4 (3)	C32—C33—C34—C35	0.6 (3)
C12—C13—C14—C15	0.6 (3)	C33—C34—C35—C36	−0.7 (3)
C13—C14—C15—C10	0.9 (3)	C34—C35—C36—C31	0.1 (3)
C11—C10—C15—C14	−1.7 (3)	C32—C31—C36—C35	0.5 (3)
P1—C10—C15—C14	−179.79 (15)	P2—C31—C36—C35	−177.42 (15)
C10—P1—C16—C21	−150.60 (15)	C25—P2—C37—C42	−28.81 (19)
C4—P1—C16—C21	87.64 (16)	C31—P2—C37—C42	−147.34 (16)
C1—P1—C16—C21	−33.46 (17)	C22—P2—C37—C42	94.13 (18)
C10—P1—C16—C17	36.28 (17)	C25—P2—C37—C38	154.75 (16)
C4—P1—C16—C17	−85.48 (16)	C31—P2—C37—C38	36.22 (19)
C1—P1—C16—C17	153.41 (15)	C22—P2—C37—C38	−82.31 (18)
C21—C16—C17—C18	−0.7 (3)	C42—C37—C38—C39	0.1 (3)
P1—C16—C17—C18	172.39 (15)	P2—C37—C38—C39	176.59 (17)
C16—C17—C18—C19	0.4 (3)	C37—C38—C39—C40	0.7 (3)
C17—C18—C19—C20	0.7 (3)	C38—C39—C40—C41	−0.4 (4)
C18—C19—C20—C21	−1.5 (3)	C39—C40—C41—C42	−0.6 (3)
C19—C20—C21—C16	1.2 (3)	C40—C41—C42—C37	1.4 (3)
C17—C16—C21—C20	−0.1 (3)	C38—C37—C42—C41	−1.1 (3)
P1—C16—C21—C20	−173.14 (14)	P2—C37—C42—C41	−177.50 (15)
