# Supplementary figures and images for: Crystal structure of bis­(acetonyltri­phenyl­phospho­nium) tetra­chlorido­cobaltate(II)
Source: Acta Crystallogr E Crystallogr Commun. 2015 Nov 4;71(Pt 12):m209–10. doi: 10.1107/S2056989015019180 (PMC4719837; doi:10.1107/S2056989015019180)

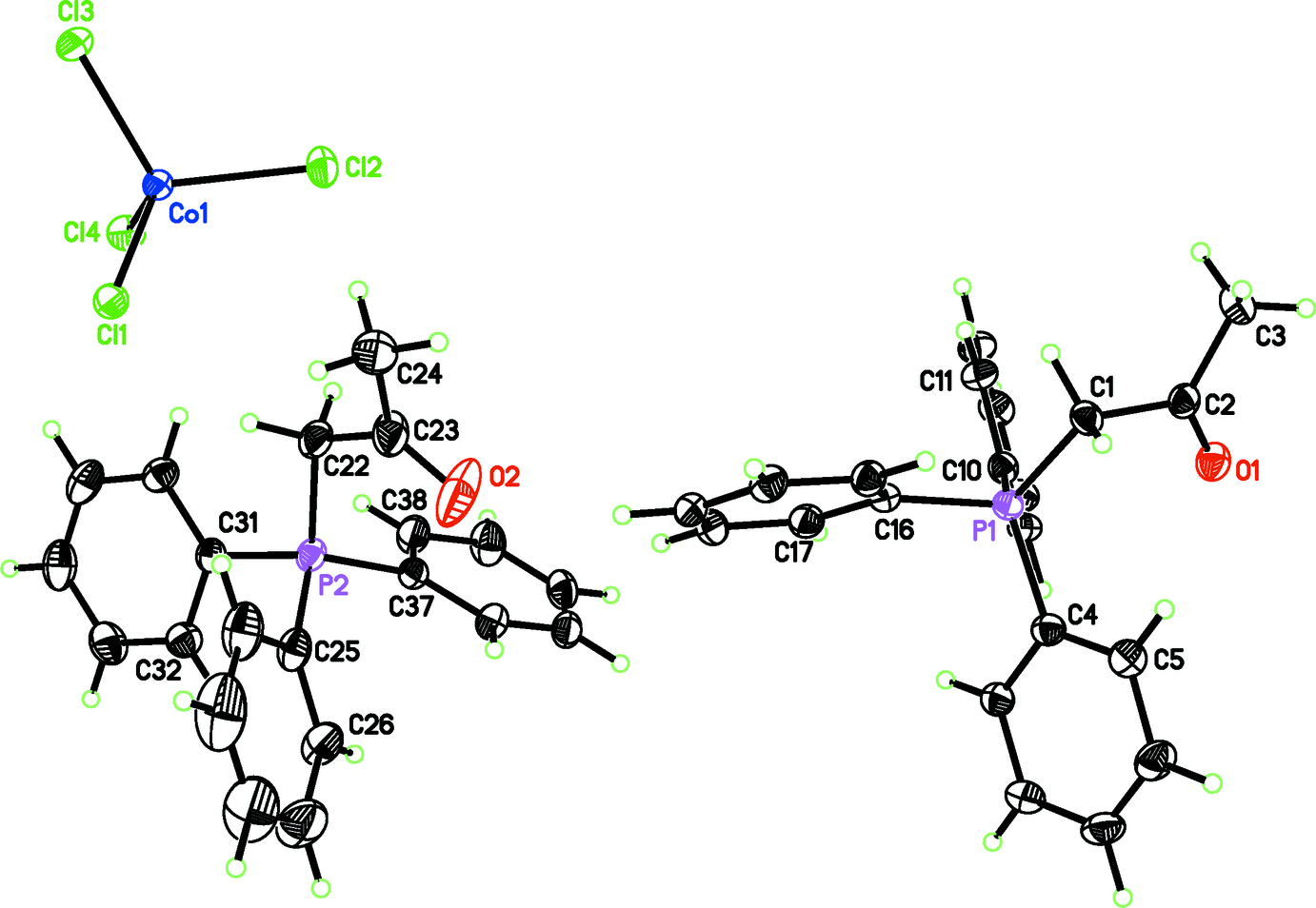

Supplement: Supplementary file 3 [file e-71-0m209-fig1.tif]

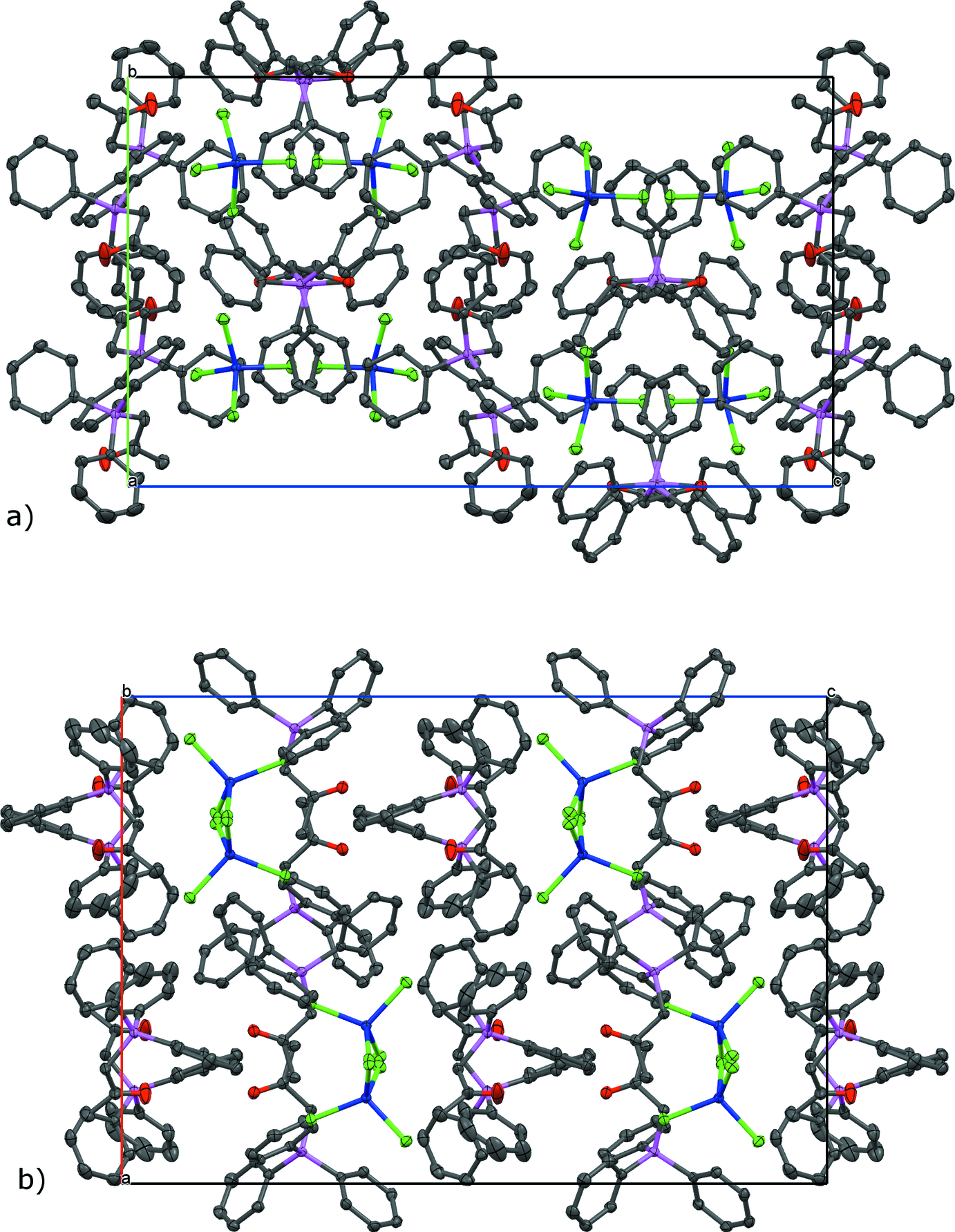

Supplement: Supplementary file 4 [file e-71-0m209-fig2.tif]
